# A Rare Autopsy Finding of Müllerian and Renal Agenesis Linked to Suspected AMHR2 Pathway Disruption

**DOI:** 10.7759/cureus.96023

**Published:** 2025-11-03

**Authors:** Zoë Rushetsky, Mariah Smith, Marcel Castor

**Affiliations:** 1 Department of Osteopathic Medicine, Philadelphia College of Osteopathic Medicine, Suwanee, USA; 2 Forensic Pathology, Dekalb County Medical Examiner's Office, Decatur, USA; 3 Forensic Pathology, Dekalb County Medical Examiner’s Office, Decatur, USA

**Keywords:** amhr2, anti-müllerian hormone, mesonephric ducts, mrkh syndrome, müllerian agenesis, paramesonephric ducts, renal agenesis

## Abstract

We present a rare incidental finding of Mayer-Rokitansky-Küster-Hauser (MRKH) syndrome identified during the autopsy of a reproductive-aged woman who died in a motor vehicle accident. In situ examination showed the absence of both the uterus and cervix, with the vaginal canal ending in a blind pouch that measured 7.0 × 6.0 cm, with a depth of 4 cm. Both ovaries were present and positioned high near the iliac vessels, demonstrating multiple cysts upon cut surfaces. The bilateral fallopian tubes were absent in their entirety. The right kidney, along with its hilar vessels and ureter, was also absent, while the left kidney and its associated structures were present but slightly enlarged. The bilateral adrenal glands were normally positioned and grossly unremarkable. The combination of absent uterus, cervix, and right kidney, along with ascended ovaries, is consistent with a developmental abnormality involving the anti-Müllerian hormone (AMH) pathway and potential disruption of the *AMHR2* gene. Recognition of these anomalies in an adult female contributes to a deeper understanding of congenital reproductive and renal malformations in females. It highlights the possible role of the AMH pathway in their embryologic development.

## Introduction

Müllerian agenesis, also known as Mayer-Rokitansky-Küster-Hauser (MRKH) syndrome, refers to the congenital absence of the uterus, cervix, and the upper two-thirds of the vagina in phenotypic females with normal ovarian function and secondary sexual characteristics. This syndrome is often diagnosed in adolescence during evaluation for primary amenorrhea [1]. Epidemiologically, MRKH syndrome is rare. Population-based studies estimate a birth prevalence of approximately 1 in 4,982 to 1 in 5,000 live female births [1]. MRKH syndrome is divided into two subtypes: type I and type II. In type I, the anomaly is isolated to the reproductive tract and is more common. Type II is associated with renal, skeletal, or auditory anomalies [2]. Alterations in the anti-Müllerian hormone (AMH) signaling pathway, particularly involving mutations in the *AMHR2* gene, can affect normal reproductive tract formation. The *AMHR2* receptor plays a key role during embryonic development. AMH binds to receptors on the mesenchyme surrounding the Müllerian ducts. This interaction initiates a SMAD-mediated process that causes the ducts to regress in male embryos. When this pathway is disrupted, through loss-of-function mutations in *AMH* or *AMHR2*, the Müllerian ducts fail to regress, resulting in persistent Müllerian duct syndrome in 46,XY individuals [3,4]. We present a case of a 27-year-old woman who had no past medical history and was found at autopsy to have findings consistent with Müllerian agenesis, renal agenesis, and abnormal ovarian positioning.

## Case presentation

**Figure 1:**
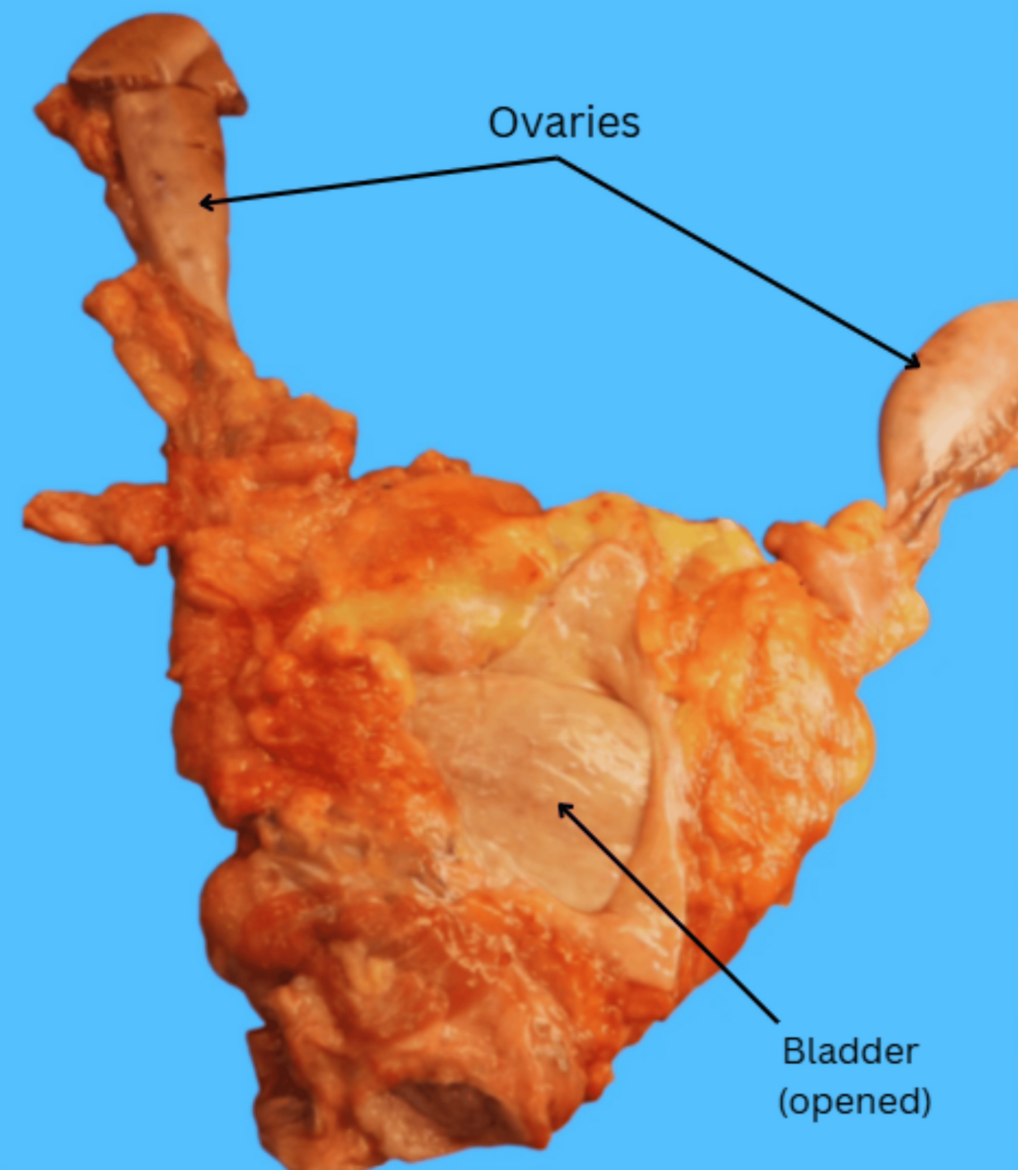
Bladder present with attached bilateral ovaries and without an associated uterus.

**Figure 2:**
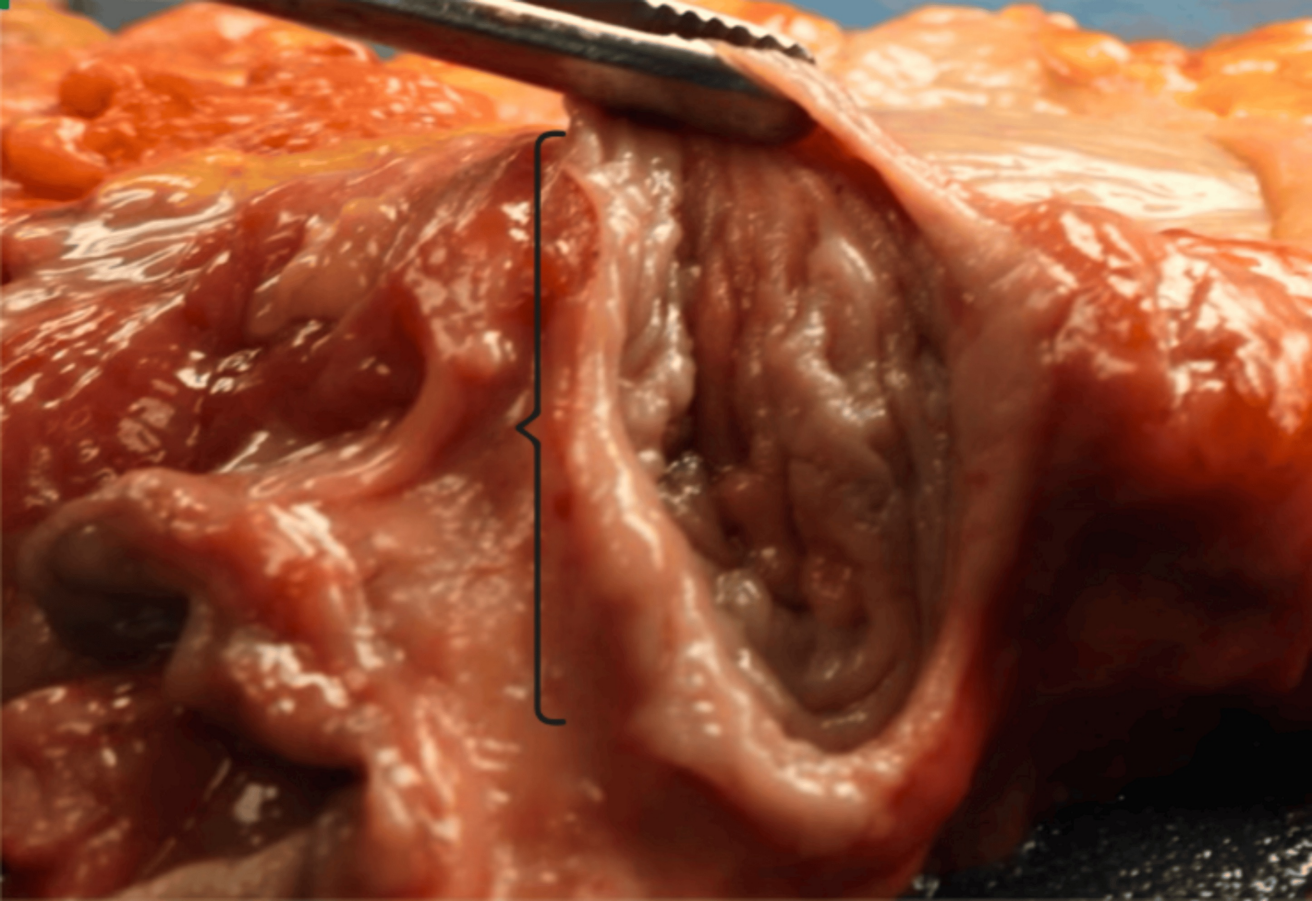
Partial vaginal agenesis with a blind-ending canal (shown above in brackets).

During an autopsy, a 27-year-old woman presented with phenotypically normal external female genitalia. However, internal examination of the pelvis demonstrated that the uterus and cervix were grossly absent (Figure 1). Additionally, the vaginal canal ended in a blind pouch that measured 7.0 x 6.0 cm, with a total depth of 4 cm (Figure 2).

**Figure 3:**
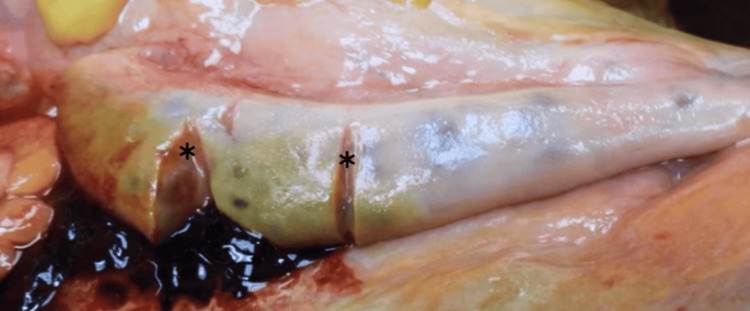
Enlarged right ovary with cystic features. *Cystic features upon sectioning.

**Figure 4:**
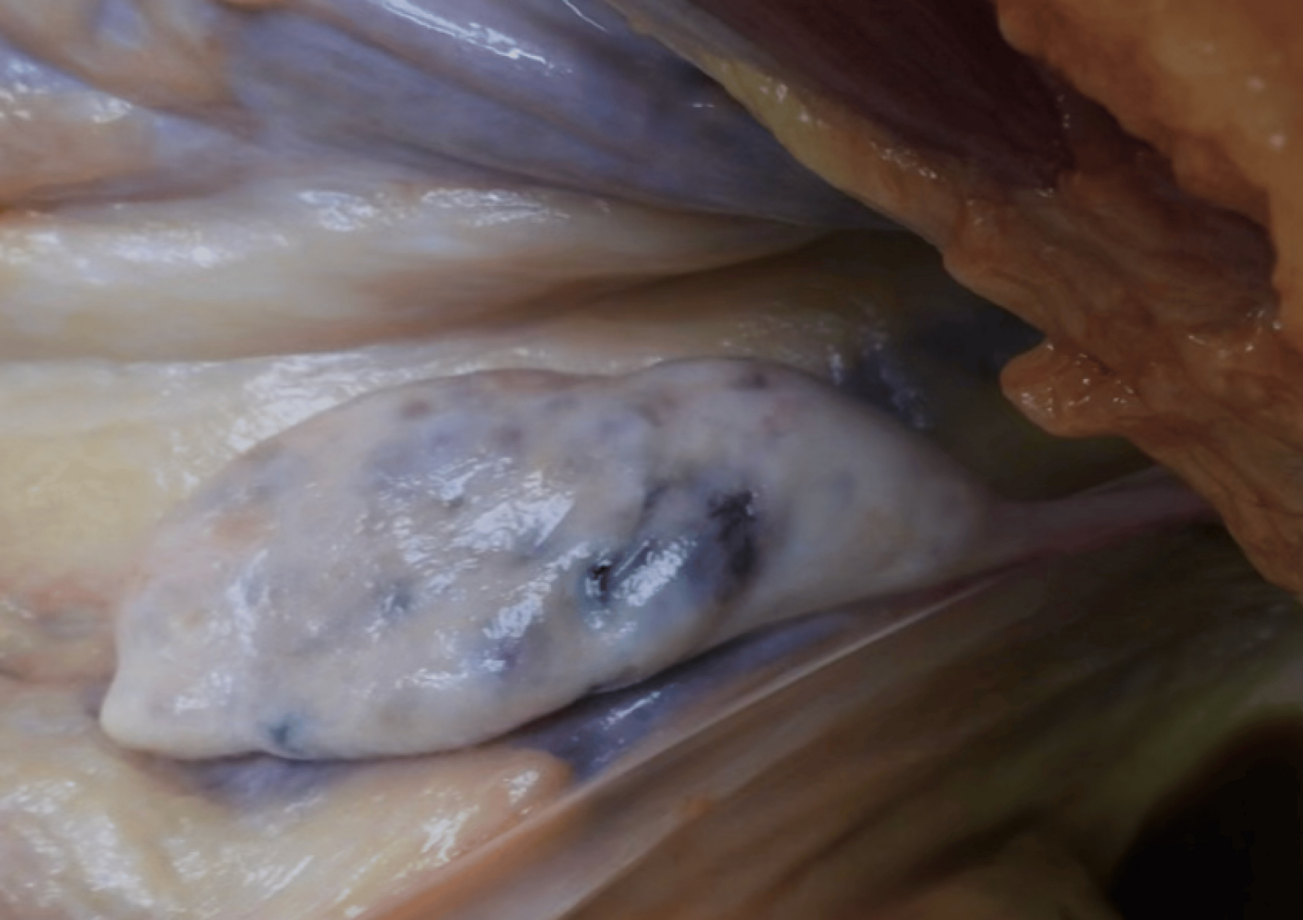
Grossly observed position of the left ovary near the bifurcation of the iliac vessels.

The bilateral ovaries were present, grossly enlarged, and upon sectioning exhibited polycystic spaces containing clear gelatinous and serous fluid (Figure 3). The ovaries were located unusually high within the pelvis, just adjacent to the bifurcation of the common iliac vessels (Figure 4). The entirety of the bilateral fallopian tubes was not grossly observed, which is consistent with the embryological nature. Medical records were not available at the time of autopsy.

Evaluation of the renal system revealed a complete absence of the right kidney, renal artery, renal vein, and ureter. However, the left kidney was present, weighing 150 grams and having an intact and smooth capsule. The left kidney was slightly hypertrophied, but no gross abnormalities were identified upon sectioning. The bilateral adrenal glands were present in their usual anatomic positioning and were grossly unremarkable.

## Discussion

This case represents a rare instance of Müllerian agenesis discovered at autopsy in a 27-year-old woman. The gross absence of the uterus and cervix, together with a blind-ending vaginal pouch, is a classic feature of Müllerian agenesis. The additional findings of ascended ovaries and right renal agenesis suggest a broader congenital defect, potentially involving signaling pathways mediated by AMH [1].

During early fetal development, both the mesonephric (Wolffian) and paramesonephric (Müllerian) ducts arise from the intermediate mesoderm along the urogenital ridge and form in proximity. Because they develop closely and simultaneously, abnormalities in one ductal system are often accompanied by defects in the other [5,6]. In male development, the mesonephric ducts remain and form the epididymis, vas deferens, and seminal vesicles, while the paramesonephric ducts regress under the influence of AMH. In female development, the paramesonephric ducts normally develop into the fallopian tubes, uterus, and upper vagina. Failure of this process results in Müllerian agenesis, which may occur alone or in combination with other anomalies [7].

MRKH syndrome, particularly type II, represents a form of Müllerian agenesis and is associated with renal agenesis or dysplasia in up to 40% of cases [2]. Given the close developmental relationship between the reproductive and urinary tracts, disruptions in genetic or hormonal signaling, such as the AMH pathway, can affect both systems [8]. This shared embryological origin helps explain the frequent renal anomalies observed in MRKH syndrome.

In this case, the ovaries were positioned high near the iliac vessels. This likely reflects the absence of the mesovarium, which normally supports and anchors the adnexa [9]. This positional abnormality, although not commonly reported, is consistent with developmental disorganization in the absence of a proper uterine scaffold.

Most cases of MRKH syndrome are diagnosed in adolescence during evaluation of primary amenorrhea. However, individuals without symptoms or a fertility evaluation may remain undiagnosed into adulthood. Symptomatic patients often present with dyspareunia (pain during sexual intercourse), which can vary depending on the depth of the vaginal pouch [10].

Since this case was identified incidentally during a postmortem death investigation due to a fatal motor vehicle collision, no clinical investigations, laboratory tests, or imaging studies were available. The diagnosis of Müllerian agenesis was established based on gross anatomical findings presented above that we observed during autopsy.

## Conclusions

This case provides a rare look at an unexpected congenital anomaly found during an autopsy. The absence of the uterus, cervix, and right kidney, as well as bilateral ascended ovaries, suggests a problem with the AMH pathway, which may involve an *AMHR2* gene mutation. Although genetic testing was unavailable at the time of autopsy for confirmation, the anatomical findings were strongly suggestive of MRKH type II or a related variant. This case expands upon our understanding of how these developmental disorders may appear in adult females. Future genetic analyses could clarify the role of *AMHR2* mutations in similar presentations.
